# Whole-Genome Sequencing-Based Characterisation of *Salmonella* Saintpaul Isolates from Human and Food Sources in Singapore: Sequence Types, Antimicrobial Resistance and Plasmid Profiles

**DOI:** 10.3390/microorganisms14091990

**Published:** 2026-09-08

**Authors:** Pandora Han, Yen Ching Lim, Wei Ching Khor, Hong Ming Glendon Ong, Matthias Maiwald, Timothy Barkham, Tse Hsien Koh, Joanna Khoo, Joanne Sheot Harn Chan, Kyaw Thu Aung

**Affiliations:** 1 National Centre for Food Science, Singapore Food Agency, Singapore 609919, Singapore; pandora_han@sfa.gov.sg (P.H.); lim_yen_ching@sfa.gov.sg (Y.C.L.); khor_wei_ching@sfa.gov.sg (W.C.K.); glendon_ong@sfa.gov.sg (H.M.G.O.); joanna_khoo@sfa.gov.sg (J.K.); chan_sheot_harn@sfa.gov.sg (J.S.H.C.); 2 KK Women’s and Children’s Hospital, Singapore 229899, Singapore; matthias.maiwald@singhealth.com.sg; 3 Duke-NUS Medical School, National University of Singapore, Singapore 169857, Singapore; koh.tse.hsien@singhealth.com.sg; 4 Yong Loo Lin School of Medicine, National University of Singapore, Singapore 117545, Singapore; 5 Tan Tock Seng Hospital, Singapore 308433, Singapore; timothy.barkham@nhghealth.com.sg; 6 Singapore General Hospital, Singapore 169856, Singapore; 7 Department of Food Science & Technology, National University of Singapore, Singapore 117542, Singapore; 8 School of Biological Sciences, Nanyang Technological University, Singapore 637551, Singapore

**Keywords:** *Salmonella enterica* subsp. *enterica* serovar Saintpaul, antimicrobial resistance, food, human, surveillance

## Abstract

*Salmonella* Saintpaul is a foodborne pathogen of public health relevance. This study characterised 79 *S.* Saintpaul isolates from human clinical and food sources collected in Singapore from 2015 to 2017. Isolates belonged to ST3602 (91.1%, *n* = 72), ST27 (6.33%, *n* = 5), ST49 (1.27%, *n* = 1) or ST50 (1.27%, *n* = 1). ST3602 predominated in both human (92.5%) and food (89.7%) isolates. Human and food isolates showed the highest resistance to penicillins and beta-lactam/beta-lactamase inhibitor combinations. The analysis identified beta-lactam resistance genes (*bla*_CTX-M-1_, *bla*_TEM-1D_), aminoglycoside resistance genes (*aph(4)-Ia*, *aac(3)-IVa*, *aadA*), and fluoroquinolone resistance genes (*qnr*S) as the most prevalent. Notably, 31.6% of isolates identified as extended-spectrum beta-lactamase (ESBL)-producing and all ESBL-positive isolates exhibited multidrug resistance. Plasmids were predicted in 75.9% (*n* = 60) of the isolates, with IncI1-I(Alpha) being the most common incompatibility group and showing a strong association with *bla*_CTX-M-1_ (Fisher’s exact test, *p* < 0.001; Φ = 0.913). Single-nucleotide polymorphism (SNP) analysis revealed close genetic relatedness between chicken and human isolates at relatively low SNP distances (0–16 SNPs), suggesting potential shared transmission pathways. This study provides epidemiological insights and antimicrobial resistance landscape of *S.* Saintpaul in Singapore, informing strategies for food safety and public health surveillance.

## 1. Introduction

*Salmonella*, a Gram-negative, facultative anaerobic bacterium belonging to the family *Enterobacteriaceae*, is one of the most significant foodborne pathogens worldwide [1]. The transmission of *Salmonella* primarily occurs through ingestion of contaminated food products of animal origin, particularly poultry, pork, and eggs. *Salmonella* infection in humans is associated with poor food hygiene, improper food storage, and inadequate heat treatment [2]. The socioeconomic impact of *Salmonella* infections extends beyond immediate health concerns, encompassing costs related to contaminated food disposal, trade restrictions, and productivity losses, with the full extent of this burden on global health and development remaining largely unknown [3].

In Singapore, non-typhoidal *Salmonella* (NTS) species are one of the most common causative agents of foodborne illness [4]. From 2001 to 2010, NTS salmonellosis accounted for more than half of reported food-borne and water-borne diseases in Singapore [5]. Recognising this burden, NTS salmonellosis became a notifiable disease in 2008, requiring medical practitioners and laboratories to report laboratory-confirmed cases within 72 h [6]. The incidence of non-typhoidal salmonellosis in Singapore has risen steadily since mandatory reporting began in 2008, increasing from 14.8 per 100,000 population in 2008 to 39.4 per 100,000 in 2016, with cases showing a particularly sharp increase from 2018 to 2019 [7].

*S.* Saintpaul is a notable non-typhoidal *Salmonella* serovar and has been associated with major outbreaks. In the United States, it is among the top 20 most common serovars detected [8]. It has been responsible for numerous large-scale foodborne outbreaks internationally. Notable incidents in the United States include the 2008 jalapeño pepper outbreak linked to contaminated produce from Mexico, which affected 1442 individuals across 43 states [9]; the 2009 alfalfa sprout outbreak involving 235 cases across 14 states [10]; and the 2013 cucumber outbreak affecting 84 people in 18 states [11]. More recently, a 2026 multistate outbreak of *S*. Saintpaul linked to backyard poultry was reported in the United States, where of 210 patients who specified backyard poultry contact, 83% reported contact with chickens and 48% with ducks [12]. Beyond the United States, *S.* Saintpaul has also been implicated in outbreaks associated with beansprouts in the United Kingdom [13] and cantaloupes in Australia [14], demonstrating its global distribution and association with fresh produce contamination. *S.* Saintpaul has also been identified in poultry, accounting for 4.2% of *Salmonella* isolates recovered from broiler litter, diet and faeces in poultry broilers [15]. Its association with turkey has further been documented, with 33% (130/391) of multidrug-resistant (MDR) *S.* Saintpaul strains linked to a 2018 multistate outbreak involving raw turkey products in the United States [16]. These incidents collectively highlight the risk posed by *S.* Saintpaul contamination across diverse food sources, from fresh produce to poultry, during production and processing stages.

In Singapore, *S.* Saintpaul has gained particular attention as it was reported for the first time in 2015 and 2016 to be among the top ten most common serovars in both human and food samples [7]. A study in Singapore detected *S.* Saintpaul as the most frequently isolated serovar from raw chicken meat [17]. This is noteworthy given its potential for asymptomatic carriage in poultry and the possibility of transmission to humans. A study from Singapore reported that more than half of *S.* Saintpaul isolates were resistant to third-generation cephalosporins in 2015 and 2016 [18]. This underscores the importance of analysing the antimicrobial susceptibility profiles of *S.* Saintpaul, for the identification of alternative antimicrobials to treat salmonellosis.

Despite its rising prominence, research on *S.* Saintpaul remains limited, particularly regarding its integrated analysis of antimicrobial resistance profiles and genetic characteristics of isolates across human and food sectors. This study aimed to determine the antimicrobial susceptibility of 79 *S.* Saintpaul isolates in Singapore, characterise their dominant sequence types, plasmid profiles, antimicrobial resistance patterns and investigate phylogenetic relationships and potential transmission pathways. Findings from this study can enhance understanding of molecular epidemiology and antimicrobial resistance patterns in *S.* Saintpaul to inform strategies for integrated food safety and foodborne disease surveillance and targeted risk management measures for food safety and public health.

## 2. Materials and Methods

### 2.1. Collection of S. Saintpaul Isolates

The study comprised the complete collection of *S.* Saintpaul isolates (*n* = 79) available from human (*n* = 40) and food (*n* = 39) sources during the period 2015–2017 in Singapore, representing all convenience samples of this serotype identified across food surveillance programmes and major hospital networks during the study period. The human isolates were obtained from bacterial biobanks containing isolates from inpatients diagnosed with foodborne salmonellosis, contributed by KK Women’s and Children’s Hospital, Singapore General Hospital, and Tan Tock Seng Hospital—three major public hospitals in Singapore. Extra-intestinal isolates (*n* = 12) were defined as those recovered from non-gastrointestinal clinical sites, specifically blood (bacteraemia) and urine (urinary tract infections), in contrast to intestinal isolates (*n* = 28) which were recovered from stool cultures. Food isolates were retrieved from the archived bacterial biobanks of national food safety, animal and environmental health laboratories, as part of routine surveillance and monitoring programmes. The isolates were streaked on tryptone soya agar (Oxoid, Hamsphire, UK) to confirm purity and for further characterisation.

### 2.2. Phenotypic Antimicrobial Susceptibility Testing

The minimum inhibitory concentration (MIC) of different antimicrobials was determined using the broth microdilution method. MicroScan Neg MIC Panel Type 44 (Beckman Coulter, Inc., Brea, CA, USA) was used according to the manufacturer’s instructions. The antimicrobial susceptibility profiles of each isolate were determined according to the available MIC interpretations published by the Clinical and Laboratory Standards Institute (CLSI) or European Committee on Antimicrobial Susceptibility Testing (EUCAST) as appropriate. To avoid overestimation of resistance, isolates with MICs in the sensitive and intermediate ranges were considered susceptible. Isolates that were resistant to three or more antimicrobial classes were classified as multi-drug-resistant strains.

All isolates were determined for their antimicrobial susceptibility to 28 antimicrobials belonging to 13 antimicrobial classes: amikacin; amoxicillin/clavulanate; ampicillin/sulbactam; ampicillin; aztreonam; cefepime; cefotaxime; cefoxitin; ceftazidime; cefuroxime; chloramphenicol; ciprofloxacin; colistin; doripenem; ertapenem; fosfomycin; gentamicin; imipenem; levofloxacin; meropenem; minocycline; nitrofurantoin; norfloxacin; piperacillin/tazobactam; piperacillin; tetracycline; tobramycin; trimethoprim/sulfamethoxazole. Extended-spectrum beta-lactamase (ESBL) enzyme production was confirmed using a combination of cefotaxime/clavulanate and ceftazidime/clavulanate.

### 2.3. Whole-Genome Sequencing (WGS) and Analysis

Whole-genome sequencing was performed by the Genome Institute of Singapore. Briefly, a single colony of each isolate grown on tryptone soya agar was inoculated into 5 mL of Universal Pre-enrichment Broth (Acumedia, Lansing, MI, USA) and incubated overnight at 37 °C. Following incubation, 1 mL of each *S.* Saintpaul overnight culture was centrifuged. An enzymatic lysis buffer was used to lyse the *S.* Saintpaul cells at 37 °C for 45 min. DNA extraction was performed with the DNeasy Blood and Tissue Kit (QIAGEN, Valencia, CA, USA) as per the manufacturer’s instructions. An M220 Focused Ultrasonicator (Covaris, Woburn, MA, USA) was used to shear genomic DNA. The NEBNext^®^ Ultra™ DNA Library Prep Kit (NEB, Ipswich, MA, USA) was used for library preparation. Sequencing of the samples was performed using a HiSeq 4000 sequencer (Illumina, San Diego, CA, USA) with 2 × 151 bp reads.

Primary sequence analysis was performed by the Genome Institute of Singapore Efficient Rapid Microbial Sequencing tool (Strain GERMS). Serotypes were predicted using the SeqSero 1.0 [19], while multi-locus sequence type (MLST) predictions were made by using SRST2 version 0.1.8 [20]. The ARGannot resistance gene database included with SRST2 was used for resistance gene prediction. Plasmid replicons were predicted using PlasmidFinder (version 2.1) [21] with assembled contigs generated by SKESA (Strategic K-mer Extension for Sequence Assembly) (version 2.5.1), which performs internal error correction optimised for Illumina short-read data without requiring a separate quality trimming step. Contigs were queried against the PlasmidFinder database (version 2.2.0). using BLASTn (version 2.16.0), applying minimum thresholds of 95% nucleotide identity and 60% coverage for hit assignment, with a replicon considered detected only if both thresholds were met simultaneously. Chromosomal mutations associated with fluoroquinolone and colistin resistance were screened using PointFinder (integrated in ResFinder version 4.7.2) [22] against the *Salmonella enterica* database, targeting the genes *gyrA*, *parC*, *pmrA*, *pmrB*, and *acrB*, with minimum coverage and identity thresholds of 60% and 90%, respectively. Whole-genome sequencing reads were analysed using a reference-based single-nucleotide polymorphism (SNP) pipeline implemented in Snippy (version 4.0.2) [23]. Paired-end reads from each *S.* Saintpaul isolate were mapped to the *S.* Saintpaul reference genome (GenBank assembly accession GCA_002290205.1, ASM229020v1), with SNPs called using a minimum coverage of 10× and minimum allele fraction of 0.9. Core genome alignment was generated using snippy-core (version 4.0.2), retaining polymorphic core sites shared across all isolates. Pairwise SNP distances were calculated using snp-dists (1.2.0) , and a maximum-likelihood phylogenetic tree was inferred from the same alignment using FastTree (version 2.2.0) under the generalised time-reversible (GTR) nucleotide substitution model.

### 2.4. Data Analysis

Phylogenetic trees were visualised and annotated in R (version 4.4.3) using the packages ape [24], treeio [25], and ggtree [26], with isolates colour-coded according to source. Statistical analyses were conducted to examine the relationships between antimicrobial resistance profiles, plasmid types, sequence types and potential invasiveness (as determined by isolation from extra-intestinal sites). These associations were evaluated using Fisher’s exact test or chi-square test, and were performed using Python (version 3.12.7), utilising the SciPy library (scipy.stats modules: fisher_exact and chi2_contingency) (version 1.13.1) [27] and the pandas library for data handling. Co-occurrence relationships between isolates and plasmid types were visualised as a chord diagram on using mpl_chord_diagram package on Python.

## 3. Results

### 3.1. Distribution of S. Saintpaul Isolates in Singapore

*S.* Saintpaul isolates in this study belonged to four sequence types, ST3602 (91.1%, *n* = 72), ST27 (6.33%, *n* = 5), ST49 (1.27%, *n* = 1) and ST50 (1.27%, *n* = 1) (Table 1). Most of the human (37/40, 92.5%) and food (35/39, 89.7%) isolates belonged to ST3602. The most common food source among ST3602 isolates was chicken (32/35, 91.4%).

Among the human isolates, 70% (28/40) were from intestinal (stool) samples and 30% (12/40) were from extra-intestinal samples (Table 1). All extra-intestinal samples belonged to ST3602, while intestinal samples belonged to ST27 (3/28, 10.7%) and ST3602 (25/28, 89.3%). Although all extra-intestinal isolates were ST3602, Fisher’s exact test showed no statistically significant association between sequence type and body site (*p* = 0.54), likely reflecting the low statistical power resulting from the small number of ST27 isolates (*n* = 3) in the dataset rather than a true absence of association.

Of the 39 food isolates, 33 (84.6%) were from chicken meat, and the rest were from duck meat (*n* = 4), pork meat (*n* = 1) and bean sprouts (*n* = 1). A total of 32 out of 33 (97.0%) chicken isolates belonged to ST3602, while the remaining belonged to ST50. Duck isolates belonged to ST27 (*n* = 2) and ST3602 (*n* = 2). Pork meat and bean sprouts isolate belonged to ST49 and ST3602, respectively.

### 3.2. Phenotypic Antimicrobial Resistance

All *S.* Saintpaul isolates in this study were susceptible to amikacin, ciprofloxacin, levofloxacin, doripenem, ertapenem and meropenem. Four human isolates (intestinal, *n* = 3; extra-intestinal, *n* = 1) and eight food isolates (chicken meat, *n* = 7; bean sprouts, *n* = 1) were susceptible to all antimicrobials tested. Most of the human (34/40, 85%) and food (23/39, 59%) isolates were resistant to at least one antimicrobial tested.

**Figure 1:**
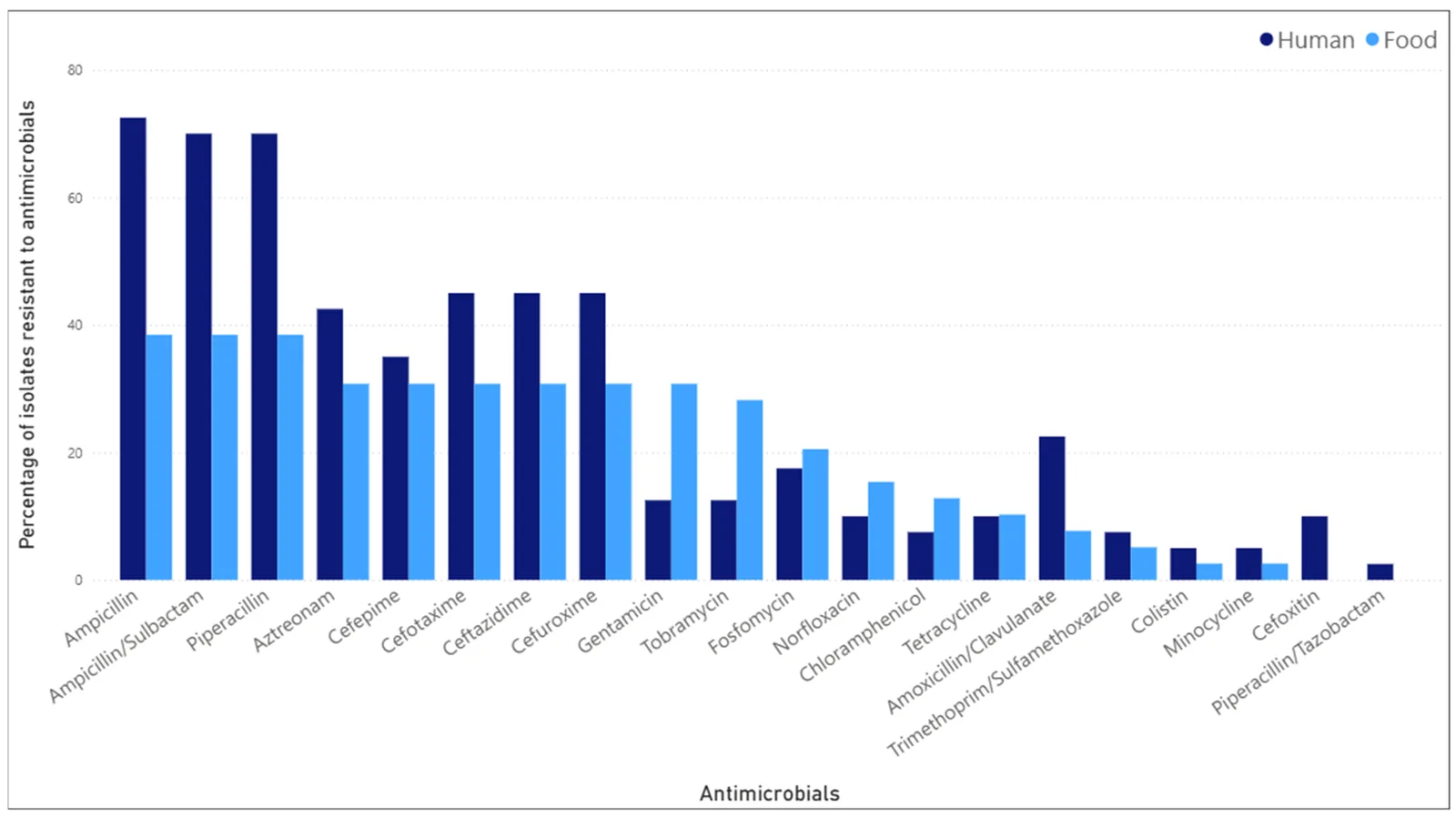
Phenotypic antimicrobial resistance in *S.* Saintpaul, comparing human and food isolates, ranked in descending order of resistance prevalence among human isolates.

**Figure 2:**
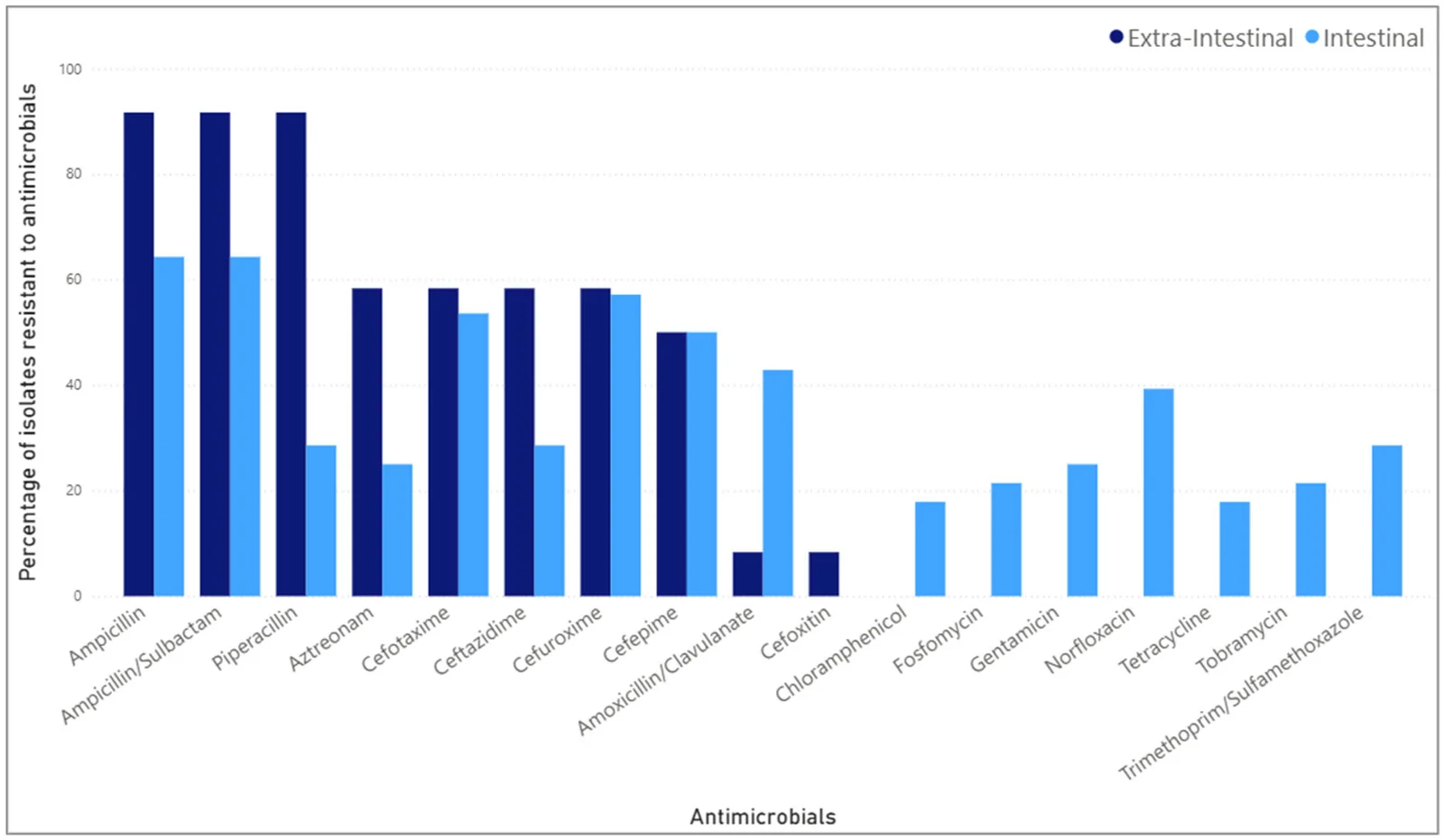
Phenotypic antimicrobial resistance profiles of *S.* Saintpaul isolates, comparing human intestinal and extra-intestinal sources, ranked in descending order of resistance prevalence among extra-intestinal isolates.

Susceptibility data are shown in Figure 1 and Figure 2. The highest resistance rates were observed for penicillins (ampicillin, 55.7%; piperacillin, 54.4%) and beta-lactam/beta-lactamase inhibitor combinations (ampicillin/sulbactam, 54.4%), with human isolates showing higher resistance than food isolates. Among human isolates, piperacillin resistance was significantly higher in extra-intestinal compared to intestinal isolates (91.7% vs. 28.6%; OR = 27.50, 95% CI: 3.03–249.49, *p* = 0.001). Extra-intestinal isolates also showed higher but non-significant resistance to ampicillin (91.7% vs. 64.3%; OR = 6.11, 95% CI: 0.69–54.51, *p* = 0.124), ampicillin/sulbactam (91.7% vs. 64.3%; OR = 6.11, 95% CI: 0.69–54.51, *p* = 0.124), ceftazidime (58.3% vs. 28.6%; OR = 3.50, 95% CI: 0.85–14.34, *p* = 0.091), aztreonam (58.3% vs. 25.0%; OR = 4.20, 95% CI: 1.00–17.57, *p* = 0.071), cefotaxime (58.3% vs. 53.6%; OR = 1.21, 95% CI: 0.31–4.76, *p* = 1.00), and cefuroxime (58.3% vs. 57.1%; OR = 1.05, 95% CI: 0.27–4.13, *p* = 1.00). Conversely, extra-intestinal showed lower resistance compared to intestinal isolates for amoxicillin/clavulanate (8.3% vs. 42.9%; OR = 0.12, 95% CI: 0.01–1.07, *p* = 0.063), norfloxacin (0.0% vs. 39.3%; OR = 0.06, 95% CI: 0.003–1.13, *p* = 0.017), aminoglycosides (gentamicin: 0.0% vs. 25.0%, OR = 0.11, 95% CI: 0.006–2.18, *p* = 0.081; tobramycin: 0.0% vs. 21.4%, OR = 0.14, 95% CI: 0.007–2.67, *p* = 0.153), and fosfomycin (0.0% vs. 21.4%; OR = 0.14, 95% CI: 0.007–2.67, *p* = 0.153), with norfloxacin reaching statistical significance. Statistical comparisons were performed using the chi-square test when all expected cell counts were ≥five, and Fisher's exact test otherwise; 95% CIs for odds ratios were estimated using the Woolf logit method, with Haldane-Anscombe correction applied where zero-count cells were present. Two human intestinal and one chicken ST3602 isolate (3/79, 3.8%) were phenotypically resistant to colistin.

Overall, 30 MDR isolates (resistant to ≥three antibiotic classes) were detected, with a higher proportion in human (60.0%, 24/40) than food sources (41.0%, 16/39). MDR prevalence was similar between intestinal (60.7%, 17/28) and extra-intestinal isolates (58.3%, 7/12). Among food isolates, MDR was observed in 43.2% of poultry samples, including chicken (42.4%, 14/33) and duck (50%, 2/4). All MDR isolates are found to be ST3602. Twenty-five (31.6%) ESBL-positive isolates were identified from chicken (*n* = 10), intestinal (*n* = 7), extra-intestinal (*n* = 6), and duck (*n* = 2) samples, all of which were multidrug-resistant.

### 3.3. Genotypic Antimicrobial Resistance and Predicted Plasmid

Among the 79 *S.* Saintpaul isolates, beta-lactam (54.4%), aminoglycoside (24.1%), and fluoroquinolone (19%) resistance genes were most frequently detected (Table 2). *bla*_CTX-M-1_, a beta-lactam resistance gene, was the most common resistance marker found (22/79, 27%). It was the most detected gene in both human intestinal (8/28, 28.6%) and human extra-intestinal (6/12, 50%) isolates. *bla*_TEM-1D_ (17/79, 21.5%) was another prevalent beta-lactam resistance gene identified. The most common aminoglycoside resistance genes were *aac(3)-IVa*, *aadA* (both 20.3%), and *aph(4)-Ia* (21.5%), with aminoglycoside resistance genes being the most detected class among chicken isolates. *qnrS* (14/79, 17.7%) was the predominant fluoroquinolone resistance gene. Chloramphenicol, tetracycline and fosfomycin resistance genes were also identified at relatively lower rates (11.4%, 11.4% and 6.3%, respectively).

**Figure 3:**
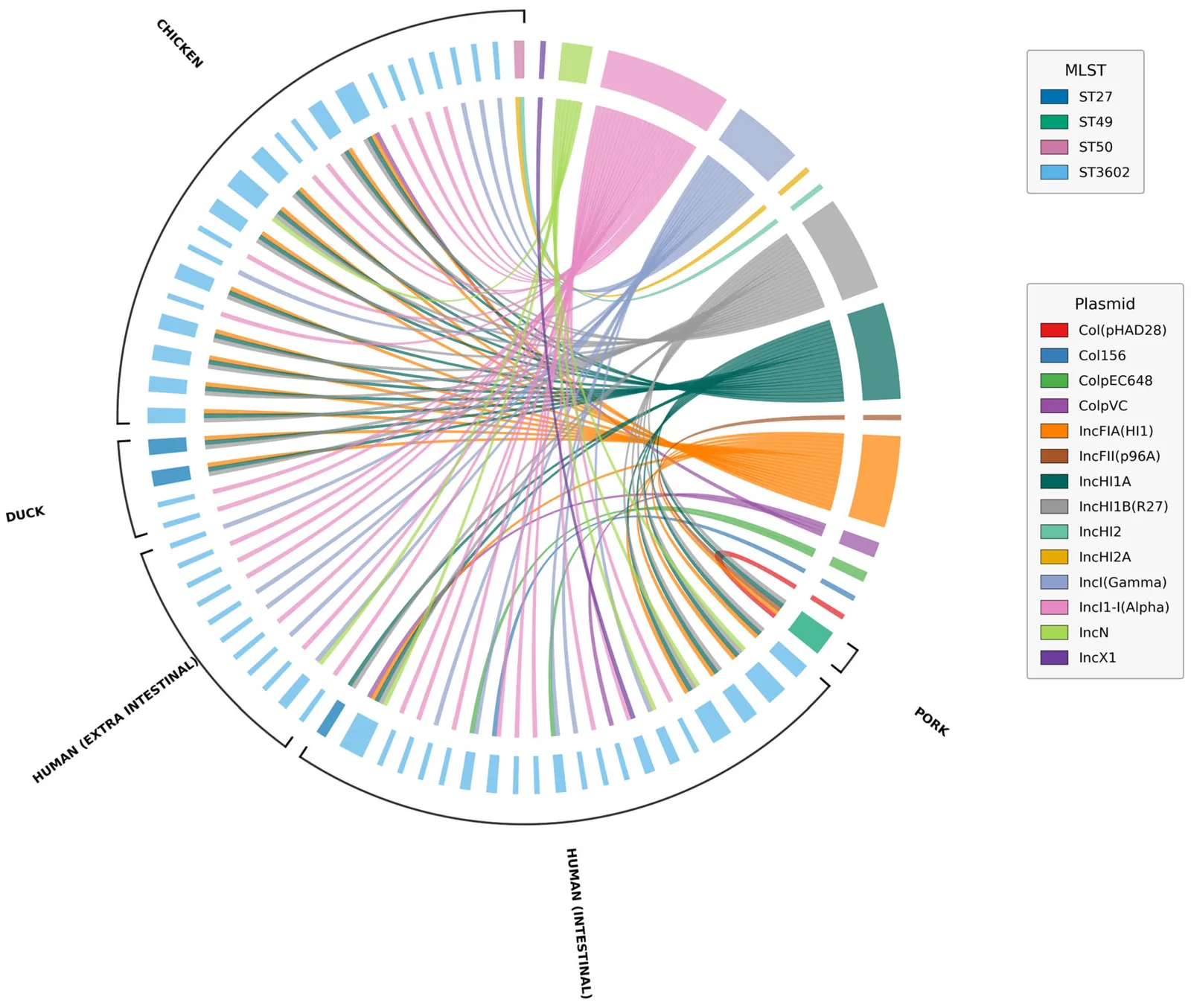
Chord diagram depicting the distribution of detected plasmid replicons among isolates. Isolates are shown on the left and connected to their corresponding plasmid replicon(s) on the right, with isolates coloured according to its sequence type and grouped by source.

Among the 79 *S.* Saintpaul isolates, 60 were found to contain at least one plasmid, and the highest detection was found for IncI1-I(Alpha) (25/60, 41.7%), IncHI1A (19/60, 31.7%), IncHI1B(R27) (19/60, 31.7%) and IncFIA(HI1) (18/60, 30%) (Figure 3). IncI1-I(Alpha) was only found in ST3602, while IncHI1A and IncHI1B(R27) were found in all ST27 and ST49 isolates and in ST3602 isolates (15/55, 27.3%). The ST50 chicken isolate had a more unique plasmid profile, harbouring IncHI2 and IncHI2A.

### 3.4. Phylogenetics Analysis

**Figure 4:**
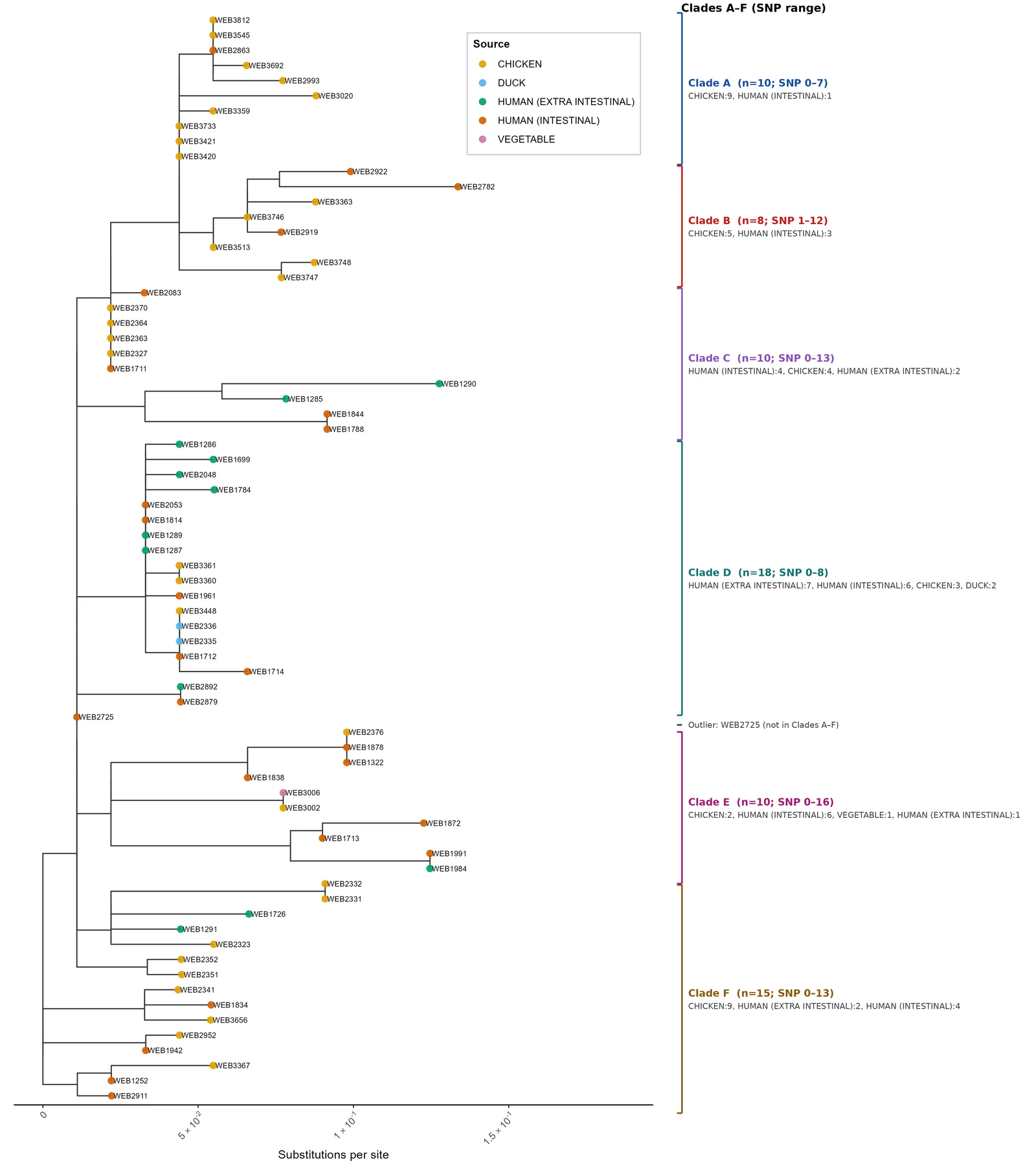
Maximum likelihood phylogenetic tree of *S.* Saintpaul ST3602 isolates constructed based on core genome alignment. Branch lengths represent substitutions per site. Each tip represents an individual isolate, coloured by isolation source as indicated in the legend.

Figure 4 shows the phylogenetic relatedness of the isolates based on core-genome SNP analysis of ST3602 isolates. Six major phylogenetic clusters were identified (Clades A–F), with within-clade pairwise SNP distances ranging from 0–16 SNPs (Figure S1). Clade A (*n* = 10; 0–7 SNPs) comprised chicken (*n* = 9) and human intestinal (*n* = 1) isolates. Clade B (*n* = 8; 1–12 SNPs) consisted of chicken (*n* = 5) and human intestinal (*n* = 3) isolates. Clade C (*n* = 10; 0–13 SNPs) contained human intestinal (*n* = 4), chicken (*n* = 4), and human extra-intestinal (*n* = 2) isolates. Clade D was the largest cluster (*n* = 18; 0–8 SNPs) and consisted predominantly of human extra-intestinal (*n* = 7) and human intestinal (*n* = 6) isolates, alongside chicken (*n* = 3) and duck (*n* = 2) isolates. Clade E (*n* = 10; 0–16 SNPs) comprised human intestinal (*n* = 6), chicken (*n* = 2), human extra-intestinal (*n* = 1), and vegetable (*n* = 1) isolates. Clade F (*n* = 15; 0–13 SNPs) contained chicken (*n* = 9), human intestinal (*n* = 4), and human extra-intestinal (*n* = 2) isolates. Human intestinal and extra-intestinal isolates were distributed across multiple clades without clear phylogenetic separation. One human intestinal isolate (WEB2725) did not cluster with any of the six main clades.

## 4. Discussion

### 4.1. Sequence Type of S. Saintpaul Isolates

ST3602 accounted for 91.1% (72/79) of *S.* Saintpaul isolates. The remaining isolates consisted of ST27 (6.3%), identified in two duck and three human intestinal samples, alongside single detections of ST50 and ST49 in pork and chicken, respectively.

Globally, *S.* Saintpaul exhibits genetic diversity across 49 sequence types, with ST50 and ST27 together accounting for 73% of 1954 genomes analysed [16]. In contrast, this study identified a predominance of ST3602, accounting for 93% (37/40) of human isolates, suggesting local clonal expansion or repeated exposure to a shared source although the underlying drivers cannot be determined from the present data alone. Similar observations have been reported in Singapore, where ST3602 was identified in 98% (48/49) *S.* Saintpaul human clinical isolates, with an average of 2.8 SNPs and a maximum of 9 SNPs between isolates, suggesting a clonal lineage [18]. To further examine within-lineage diversity and potential transmission relationships, SNP-based phylogenetic analysis was subsequently performed on ST3602 isolates in this dataset. Although isolates were distributed across multiple clades reflecting phylogenetic substructure within ST3602, the overall SNP distances remained low, consistent with a clonal lineage. This level of genomic similarity may indicate a common source or sustained exposure. However, without specific epidemiological data such as spatial-temporal and case interview data, it does not definitively establish whether transmission arose from a single contamination event or persistent contamination within the supply chain.

Among all food isolates (*n* = 39) in this dataset, 84.6% (33/39) were recovered from chicken meat. In earlier local research *S.* Saintpaul was the most prevalent serovar found in chicken meat [17]. Additionally, a study of food sources from Morocco found that *S.* Saintpaul was isolated exclusively from poultry sources, with equal distribution between chicken (50%) and turkey (50%) [28]. In this study, ST3602 was detected in food matrices across both poultry and vegetables, with 97% (32/33) of chicken isolates identified as ST3602. Similarly, a local study found that all 17 *S.* Saintpaul isolates obtained from chicken meat belonged to ST3602 [17]. However, as these isolates represent convenience samples from archived biobanks, the collection may not be fully representative of all *S*. Saintpaul cases in Singapore during this period, and potential selection bias cannot be excluded. The presence of ST3602 in both chicken and human isolates supports a potential foodborne transmission pathway, particularly given the established role of poultry as a *Salmonella* reservoir [29]. This underscores the need for targeted surveillance and control measures in the poultry sector. Lessons from the 2008 multistate *S.* Saintpaul outbreak in the United States remain relevant, where systemic challenges such as inadequate traceability, poor stakeholder communication, and delayed detection highlighted the importance of integrated surveillance programmes, robust supply chain documentation, and coordinated outbreak response frameworks across all food sectors [30].

### 4.2. Phenotypic Antimicrobial Resistance in S. Saintpaul Isolates

The similar resistance patterns observed between human and food isolates align with previous studies reporting associations between antimicrobial use in food-producing animals and comparable resistance patterns in human isolates, including evidence from Spain linking increased antimicrobial consumption in food animals with higher prevalence of resistant *Salmonella* in humans [31,32].

Although *Salmonella* infections typically cause self-limiting gastroenteritis, antimicrobial therapy is necessary in severe cases, particularly in young children and immunocompromised individuals [33]. Both human and food isolates showed the highest resistance to ampicillin, piperacillin, and ampicillin/sulbactam, consistent with resistance profiles reported in human non-typhoidal salmonellosis from China [34,35]. The lower resistance rate observed with amoxicillin-clavulanate compared to ampicillin-sulbactam may reflect the greater inhibitory potency of clavulanate against TEM- and CTX-M-type β-lactamases [36], the predominant Class A genes identified in this study (*bla*_CTX-M-1 _and *bla*_TEM-1D_).

Ampicillin, chloramphenicol, and trimethoprim-sulfamethoxazole remain traditional first-line treatments for *Salmonella* infections [1]. In this study, resistance to ampicillin was high among human isolates (72.5%), while resistance to chloramphenicol and trimethoprim-sulfamethoxazole remained low (7.5% each), suggesting that these latter two agents may remain viable treatment options. Due to the emergence of resistant strains, fluoroquinolones and extended-spectrum cephalosporins are among the remaining recommended options for treating severe salmonellosis [37]. All 79 isolates were susceptible to fluoroquinolones (ciprofloxacin and levofloxacin), consistent with previous findings in Singapore and Brazil [18,38]. In contrast, resistance to third-generation cephalosporins (cefotaxime, ceftazidime) was observed in 35–45% of human isolates using CLSI breakpoints. Although this rate is lower than the 55.1% reported in Singapore [18], it remains clinically significant given their World Health Organization (WHO) classification as Critically Important Antimicrobials of highest priority and the limited therapeutic alternatives available for invasive salmonellosis [39]. Together, this study support fluoroquinolone as the more clinically reliable option over third-generation cephalosporins for severe *S.* Saintpaul infections, though the detection of *qnrS* across 17.7% of isolates, discussed in Section 4.3, warrants consideration. Extra-intestinal isolates showed higher resistance rates to beta-lactams than intestinal isolates, mirroring *S.* Enteritidis patterns in Singapore [40], which may partly reflect prior antibiotic exposure disrupting colonic flora and selectively enriching resistant strains [41].

Among chicken isolates, ampicillin resistance (39.4%) was lower than previously reported in Singapore (88%) and Brazil (82%) [17,38], possibly reflecting differences in antibiotic usage across farms. Amoxicillin/clavulanate resistance (9.1%) was comparable to local poultry data (11.8%) [17] but lower than Brazilian poultry (25%) [38]. The similar resistance patterns between human and food isolates further support potential cross-species transmission of resistant bacteria [32].

A total of 25 isolates (31.6%) were identified as ESBL-producing, distributed across human (*n* = 13), chicken (*n* = 10), and duck (*n* = 2) sources. All ESBL-producing isolates exhibited multidrug resistance, consistent with the global expansion of CTX-M-type ESBLs and their association with MDR phenotypes mediated through mobile genetic elements [42]. The presence of resistant strains in food sources underscores the importance of restricting antimicrobial use in food-producing animals to therapeutically necessary cases as part of broader One Health efforts to mitigate antimicrobial resistance.

### 4.3. Antimicrobial Resistance Genotype and Plasmid Profiling in S. Saintpaul Isolates

The most frequently detected β-lactam resistance gene was *bla*_CTX-M_ (22/79), consistent with reports from the United Kingdom identifying CTX-M-1 as a primary resistance determinant in both human and food *Salmonella* isolates [43]. CTX-M enzymes are class A β-lactamases commonly disseminated in non-typhoidal *Salmonella* serovars through conjugative plasmid-mediated horizontal gene transfer [44,45]. These enzymes efficiently hydrolyse cefotaxime and confer resistance to penicillins and extended-spectrum cephalosporins, limiting therapeutic options for invasive salmonellosis [45]. Plasmid families such as IncI1 and IncN play a central role in the mobilisation and persistence of *bla*_CTX-M_ genes within and between human and animal bacterial populations [44,46]. The detection of CTX-M-1 in both intestinal and extra-intestinal *S.* Saintpaul isolates is of clinical concern, as it suggests broader dissemination of this enzyme within the serovar and indicates potentially reduced efficacy of extended-spectrum cephalosporins in the treatment of invasive salmonellosis [37,40]. Beyond intra-serovar dissemination, *bla*_CTX-M-1_ has been documented across multiple *Enterobacteriaceae* species in human and animal reservoirs, with IncI1-carried *bla*_CTX-M-1_ identified in poultry faecal samples and subsequently detected in *Escherichia coli* from human patients, suggesting animal-to-human transmission [47]. Human farming and food chain activities are thought to drive the dissemination of ESBL-producing *Enterobacteriaceae*, compounded by widespread antibiotic use in clinical and agricultural settings [48]. These findings highlight the role of plasmid-mediated transmission and anthropogenic pressures in driving *bla*_CTX-M-1_ dissemination across species and ecological compartments.

Among aminoglycoside resistance genes, *aadA* was notably prevalent. This gene encodes a 29 kDa protein that inactivates streptomycin through adenylation [49], but not to gentamicin or amikacin, which are of greater clinical relevance in treating severe human infections [50]. Additionally, the frequent detection of *aph(4)-Ia* and *aac(3)-IVa*, consistent with findings in human and food *S.* Infantis isolates from Switzerland [51], alongside their prevalence in chicken isolates, may suggest extensive aminoglycoside use in poultry farming.

*The qnrS* was detected in 14/79 (17.7%) isolates, representing the most frequently identified fluoroquinolone resistance gene in this study. Although *qnrS* generally confers only low-level quinolone resistance, it may facilitate bacterial survival under quinolone pressure and the subsequent selection of higher-level resistance mechanisms, including chromosomal quinolone resistance-determining regions (QRDR) mutations [52,53]. In this dataset, WGS-based chromosomal analysis identified no recognised resistance-associated substitutions at the principal *gyrA* Ser83/Asp87 or *parC* Ser80 QRDR hotspots, and no mutations were detected in *gyrB* or *parE*. Ciprofloxacin MICs were determined using the MicroScan Neg MIC panel, which has a lowest testable concentration of 0.5 µg/mL and cannot resolve the *Salmonella*-specific susceptible breakpoint of ≤0.06 µg/mL from the intermediate range of 0.12 to 0.5 µg/mL [54]. While a nalidixic acid-resistant, ciprofloxacin-susceptible phenotype has been associated with treatment failures in systemic *Salmonella* infections [55], CLSI guidelines removed nalidixic acid interpretive breakpoints for *Salmonella* spp. in M100-S26 (2016) owing to its inability to reliably predict susceptibility to fluoroquinolones used in the treatment of *Salmonella* infections, with both false-resistant and false-susceptible results reported [56]. Taken together, the phenotypic and genotypic findings demonstrate the complementary value of both approaches, with phenotypic testing characterising expressed antimicrobial susceptibility, while genomic analysis can provide insights into potential resistance mechanisms and identify determinants that may not be detected by phenotypic methods alone. Nevertheless, future studies could consider incorporating ciprofloxacin Etest or broth microdilution to achieve the resolution required for detection of reduced fluoroquinolone susceptibility in *Salmonella*.

No *mcr* genotypes were detected, despite phenotypic colistin resistance being observed in 3.8% (3/79) of *S.* Saintpaul isolates. This phenotype-genotype discordance has been similarly reported in *Salmonella* from aquaculture settings [57]. Chromosomal analysis identified no recognised mutations in *pmrA*, *pmrB*, *phoP* or *phoQ*. The absence of both plasmid-mediated and chromosomal resistance determinants suggests that the observed phenotypic resistance may be attributable to non-specific mechanisms such as porin loss or efflux pump upregulation, which can reduce colistin susceptibility independently of specific resistance determinants [58]. Although broth microdilution is the recommended method for colistin susceptibility testing [54], factors including colistin adsorption to plastic surfaces, test medium composition, cation concentration, inoculum density, and endpoint determination may influence measured MICs, and minor methodological variation cannot be completely excluded. Notably, *S.* Saintpaul has been reported as the third most prevalent *mcr*-carrying serotype among *mcr*-producing *Salmonella* globally [59], warranting monitoring for *mcr* acquisition in local population.

The most common plasmid detected was IncI1, a well-recognised vector for β-lactam resistance gene dissemination. A strong positive association was observed between IncI1-I(Alpha) and *bla*_CTX-M-1_ (Fisher’s exact test, *p* < 0.001; Φ = 0.913), indicating these determinants frequently occurred together, consistent with evidence that *bla*_CTX-M-1_ is predominantly carried on IncI1 plasmids in non-human *Salmonella* (food, livestock, food production environment, and non-food-producing animals) [60]. The conjugative transferability of *bla*_CTX-M-1_-carrying IncI1 plasmids across *Salmonella* serovars and *E. coli* suggests horizontal acquisition from other *Enterobacteriaceae* [61]. The detection of IncHI plasmids across multiple sequence types is also noteworthy, as these plasmids carry diverse resistance modules including β-lactamases organised within integrons and composite transposons [62,63], and their dissemination across bacterial lineages underscores the importance of continued genomic surveillance.

### 4.4. Phylogenetic Relationships Between Human and Food S. Saintpaul Isolates

Core-genome SNP analysis of ST3602 isolates revealed intermixing of *S.* Saintpaul from human clinical and food-associated sources across multiple clades, indicating co-clustering of genetically related strains between humans and the food chain. The distribution of isolates across clades at relatively low SNP distances reflects within-lineage diversity, consistent with the broader genomic diversity documented for *S.* Saintpaul globally, where four distinct phylogenetic groups have been identified among over 4700 isolates in the NCBI Pathogen Detection database [64]. The absence of clear phylogenetic segregation between intestinal and extra-intestinal isolates suggests that invasive disease may reflect host susceptibility, virulence determinants, exposure dose, or prior antibiotic exposure rather than distinct phylogenetic lineages, though limited sample size cannot be excluded. This contrasts with serovars such as *S.* Typhimurium ST313 in sub-Saharan Africa, where invasive isolates harbour a distinct genotype compared to gastrointestinal strains [65,66].

The clustering of poultry-associated isolates, particularly chicken, with human intestinal isolates at low SNP distances supports poultry as a potential source and transmission vehicle, consistent with previous study [34]. Pairwise SNP differences of fewer than 10 SNPs within several clades suggest recent common ancestry, a threshold similarly applied in a 2023 multinational *S.* Saintpaul outbreak linked to cantaloupe where cases clustered within six SNPs [67,68]. The co-clustering of duck isolates with human and chicken isolates highlights the potential role of multiple avian sources, while the presence of a vegetable isolate within a mixed-source clade suggests possible cross-contamination during production or handling [69].

Overall, these findings corroborate poultry as a primary source while indicating that fresh produce may also contribute to *S.* Saintpaul dissemination in Singapore. WGS integrated with epidemiological data demonstrated utility in resolving transmission pathways beyond conventional characterisation methods, and this dataset may serve as a reference for future local surveillance.

## 5. Conclusions

This study characterised 79 *S.* Saintpaul isolates from food and human sources in Singapore, revealing the predominance of ST3602 across both sources. Close genetic relatedness between chicken and human isolates at low SNP distances is consistent with a possible poultry-associated foodborne transmission pathway. The detection of ESBL-producing, multidrug-resistant ST3602 strains in both food and human isolates, alongside notable resistance to third-generation cephalosporins classified by the WHO as Critically Important Antimicrobials of highest priority, underscores the importance of antimicrobial selection in the management of invasive salmonellosis. The strong association between *bla*_CTX-M-1_ and IncI1 plasmids further suggests plasmid-mediated dissemination as a key mechanism underpinning ESBL spread within this lineage. However, the cross-sectional design, temporal data limited to year of isolation, and absence of patient clinical data, including dietary history and prior antimicrobial exposure, preclude causal inference, robust temporal signal analysis, food source attribution, and contextualisation of resistance profiles in human isolates. Prospective longitudinal studies with broader sampling scopes and larger sample sizes across the food production chain are needed to further resolve transmission dynamics and the epidemiological significance of ST3602 in human salmonellosis. Future work incorporating virulence gene profiling across clinical and food isolates would further complement these findings by providing additional insights into the pathogenic potential of *S.* Saintpaul across sources. Continued integrated genomic surveillance across human, animal, and food sectors remains essential to support source attribution, inform antimicrobial stewardship, and guide targeted interventions, ultimately to reduce the burden of salmonellosis in Singapore.

## Figures and Tables

**Table 1:** *S*. Saintpaul isolates by source and sequence types.

Source of Samples	Isolates (*n*)	ST27 (*n*)	ST49 (*n*)	ST50 (*n*)	ST3602 (*n*)
Human	40	3	0	0	37
Intestinal	28	3	0	0	25
Extra-Intestinal	12	0	0	0	12
Food	39	2	1	1	35
Chicken Meat	33	0	0	1	32
Duck Meat	4	2	0	0	2
Pork Meat	1	0	1	0	0
Bean Sprouts	1	0	0	0	1
Total	79	5	1	1	72

**Table 2:** Antimicrobial resistance genes present in *S*. Saintpaul isolates.

Resistance Genes		Total (*n* = 79)	Human		Food	
			Intestinal (*n* = 28)	Extra-Intestinal (*n* = 12)	Chicken (*n* = 33)	Non-Chicken (*n* = 6)
Beta-lactam	*bla*_CMY_	4	3	1	0	0
	*bla*_CTX-M-1_	22	8	6	6	2
	*bla*_CTX-M-2_	1	0	0	1	0
	*bla*_CTX-M-9_	3	0	0	3	0
	*bla*_DHA_	1	1	0	0	0
	*bla*_TEM-1D_	17	7	4	6	0
Aminoglycoside	*aac(3)-IVa*	16	5	0	11	0
	*aac(3)-VIa*	1	0	0	1	0
	*aadA*	16	6	0	10	0
	*aph(4)-Ia*	17	5	0	12	0
Fluoroquinolone	*qnrS*	14	5	0	9	0
	*qnrB*	1	1	0	0	0
Phenicol	*catA2*	1	1	0	0	0
	*cmlA*	1	1	0	0	0
	*floR*	8	3	0	5	0
Fosfomycin	*fosA*	5	1	0	4	0
Tetracycline	*tetA*	8	5	0	3	0
	*tetB*	1	0	0	0	1
	*tetM*	1	1	0	0	0

## Data Availability

The original data presented in the study are openly available in Sequence Read Archive (SRA) database under Bioproject accession number PRJNA1477691.

## Supplementary Materials

The following supporting information can be downloaded at: https://www.mdpi.com/article/10.3390/microorganisms14091990/s1, Figure S1: Core-genome SNP distance heatmap; Figure S2: Resistance gene clustering heatmap.
